# Detecting and Improving Human Cognitive State in Real-Time Using Data-Driven Adaptive Systems: A Systematic Review

**DOI:** 10.3390/bioengineering13070734

**Published:** 2026-06-24

**Authors:** Abhineet Rajendra Kulkarni, Pranav Madhav Kuber

**Affiliations:** 1 Department of Computer Science, University of Florida, Gainesville, FL 32611, USA; 2 Industrial and Systems Engineering Department, Rochester Institute of Technology, 1 Lomb Memorial Drive, Rochester, NY 14623, USA; pmk2015@rit.edu

**Keywords:** physiological sensing, cognitive state detection, neuromuscular system, machine learning, adaptive automation, brain–computer interface

## Abstract

Changes in human attention, workload, or alertness over time can affect task performance and may even increase the risk of injury. Detecting these changes in real time can be beneficial in improving system performance and safety. We reviewed 27 studies that developed models to sense physiological signals, classify one’s cognitive state, and deliver automated intervention. Interventions included providing real-time feedback, adjusting the task’s difficulty, or modifying automation levels across driving, education, rehabilitation, and human–robot collaboration applications. The findings showed that electroencephalography (EEG) sensors were used in 70% of studies, with attention (56%) and mental workload (26%) considered as the most targeted cognitive states. Within-subject classification reached 81.85–95.81% for multi-class tasks in laboratory settings. The most common interventions included neurofeedback display (30%) and task difficulty adjustment (19%), while automation adjustment was less frequent (11%). Only 33% of studies mentioned a latency of 15 milliseconds to 2.5 s, and all systems operated reactively by detecting cognitive states after their onset rather than anticipating them. The provided recommendations focus on the detection of multiple interacting cognitive states and predictive cognitive state trajectories. This review presents key directions for future research and provides a foundation for designing more effective cognitive state adaptive systems.

## 1. Introduction

Cognitive performance degradation among human operators is a well-documented contributor to adverse outcomes. Such fatigue-induced impairments have been identified as a probable cause or contributing factor in accidents, with drowsy driving alone accounting for approximately 100,000 crashes and 1550 fatalities annually in the United States [1,2]. Similarly, elevated error rates are seen in surgical rooms over prolonged periods [3], as well as during sustained monitoring tasks over extended shifts in industrial settings [4]. The primary cause for this is often not just the changes occurring in operators’ cognitive states, but also the lack of adaptive machinery and environments that respond to changes in reduced human capabilities.

A cognitive state is a snapshot in time of an individual’s mental processes that reflect how attention, perception, memory, and decision-making are organized in response to internal and external demands [5,6]. A cognitive state has been traditionally categorized based on vigilance and arousal, attention control, brain network states, cognitive load, experiential/metacognitive states, and affective modulation states (Table 1). Vigilance and arousal determine one’s readiness for processing and response. Attention control and brain network states then shape how information is selected and organized, shifting between external task engagement, internal mentation, and dynamic switching, driven by salience [5]. Within this system, cognitive load, experiential states, and affective modulation reflect how demands, subjective engagement, and emotion continuously influence performance and behavior. Any change in cognitive state often also leads to variations in physiological signals (like heart rate, breathing rate, skin temperature, or brain activity) [6]. These changes in physiological signals can be detected by using sensors and can ultimately help in identifying cognitive states [6]. Machine learning (ML) and deep learning algorithms have been commonly used to classify cognitive states by learning patterns from physiological, behavioral, or neurocognitive signals, such as EEG, eye tracking, heart rate variability, or performance metrics [7]. Traditional models like Support Vector Machine (SVM) and Random Forest are often applied to engineered features to distinguish states such as alertness, fatigue, and workload by finding optimal decision boundaries or combining multiple decision rules. Deep learning approaches such as Convolutional Neural Networks (CNNs) can directly learn hierarchical representations from raw or minimally processed signals [7], enabling more automated and potentially more robust classification of cognitive states through end-to-end training.

Prior studies show that physiological signals can also drive real-time task adaptation [8], and have been implemented to develop adaptive systems across diverse applications, including passive brain–computer interfaces (BCI) that monitor user state and engage interventions (from simple auditory/display notifications to electric pulses) to improve their state [9,10]. For example, the NeuroPace RNS system monitors brain waves and provides targeted electrical pulses to stop seizures in adults with medication-resistant epilepsy [11]. Such adaptive systems can also be applied across diverse applications. In automated driving, the brain activity of drivers, detected using EEG (electroencephalogram) sensors, can be utilized to detect drowsiness and adapt the level of automation accordingly to reduce errors during handover [6]. Similarly, classroom engagement can be determined by detecting attentional decline in students so as to adjust the level of difficulty or teaching style. Similarly, an adaptive exoskeleton can be developed that detects muscle activity, classifies the fatigue level, and adjusts the level of assistance provided to the user based on the task requirement [12]. Overall, such systems show great potential in improving human comfort, safety, and performance.

Prior reviews have indeed explored different aspects of adaptive systems driven by physiological signals. For instance, classification algorithms for brain–computer interfaces were reviewed (except for post-classification stages) in a review article [13]. Another study reviewed ML techniques for EEG-based workload classification across 83 studies but did not address intervention or decision frameworks [14]. Similarly, a study reviewed adaptive autonomy from a human factors perspective across 60 studies but did not address physiological sensing [15]. A recent systematic review [16] synthesized physiological methods for physical and cognitive workload estimation across 35 studies, which involved feature extraction and supervised learning models. However, the review was confined to the sensing and classification approaches and to a single construct workload. In this study, we conduct a holistic review of the articles that not only used physiological sensing for detecting cognitive states, but also developed interventions to improve cognitive states. We aimed to understand the current state of such systems and whether they can perform efficiently in real time. Thus, the objectives of this review were to (a) identify the best set of sensors for real-time cognitive state detection, (b) determine the most frequently targeted cognitive states, (c) evaluate the different types of interventions, and (d) identify potential challenges in their real-world implementation. Before conducting the review, we expected that most systems would use EEG-based sensing for binary classification, with limited real-world implementations and latency within a second from detection onset. To our knowledge, this is the first review to synthesize findings across sensing, classifications, decision frameworks, and intervention stages simultaneously. Overall, the findings presented in this article can benefit researchers by providing recommendations for the development and implementation of such closed-loop adaptive systems.

## 2. Materials and Methods

### 2.1. Search Strategy and Screening

A systematic literature search was conducted across the PubMed, Scopus, IEEE Xplore, and Web of Science databases using the PRISMA 2020 guidelines. This search included studies that were focused on closed-loop systems that combine physiological sensing with cognitive state classification and adaptive intervention. Keywords used were as follows: ((“closed-loop” OR “biocybernetic” OR “neuroadaptive” OR “neurofeedback” OR “biofeedback” OR “brain-computer interface” OR “adaptive automation”) AND (“EEG” OR “fNIRS” OR “ECG” OR “eye tracking” OR “EMG” OR “heart rate variability” OR “GSR”) AND (“cognitive state” OR “mental workload” OR “cognitive load” OR “attention” OR “fatigue” OR “engagement” OR “vigilance” OR “stress”)). The first group targeted system type, including terms for closed-loop architectures, neurofeedback protocols, and brain–computer interfaces. The second group targeted physiological sensors commonly used for cognitive state detection. The third group targeted cognitive states relevant to operational performance, including workload, attention, fatigue, and stress. Modality and system terms were restricted to title, abstract, and author keyword fields rather than searched across the full text. We acknowledge that a broader strategy that searches for physiological terms generally and verifies physiological derivation at the screening stage may have recovered additional studies. The database-specific search fields, date ranges, and filters that were applied are shown in Table 2. The complete search string, as implemented in each database, including field tags, filters, and date ranges, is provided in Supplementary Material S1, with the latest search conducted in March 2026.

**Figure 1:**
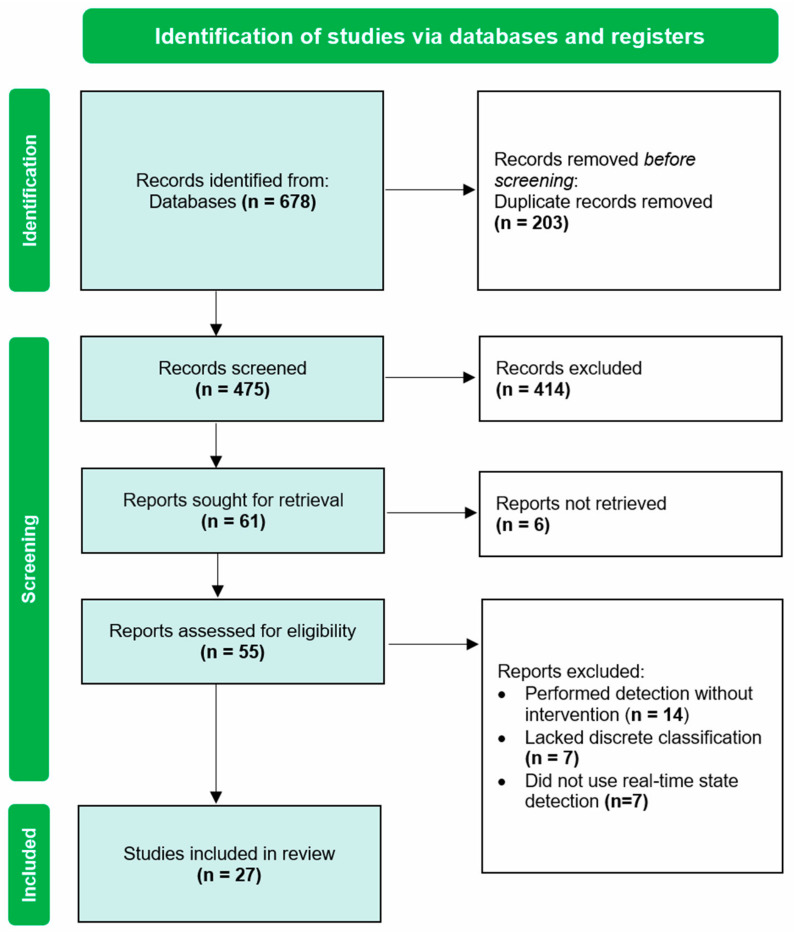
PRISMA 2020 flow diagram showing the identification, screening, eligibility assessment, and inclusion of studies.

The search yielded 678 records, as depicted in the complete PRISMA flow diagram (Figure 1). After removing 203 duplicates, 475 were screened by title and abstract. Of these, 414 were excluded. The remaining 61 records were sought for full-text assessment, with 55 being retrieved and assessed for eligibility. The final article pool comprised 27 studies that met all four inclusion criteria. This review was not pre-registered on PROSPERO. Title and abstract screening were conducted independently by both reviewers, and full-text assessment was likewise performed independently. Disagreements at either stage were resolved by discussion until a consensus was reached.

### 2.2. Eligibility Criteria

Studies were included if they implemented a system meeting all four criteria (physiological sensing, cognitive state classification, decision framework, and adaptive intervention) and targeted at least one cognitive state. Studies were excluded if they performed detection without intervention, used only subjective measures, or addressed acute clinical therapeutics. Studies were retained if the primary adaptation target was a cognitive state (attention, workload, fatigue), even when the clinical population had a neurological condition.

### 2.3. Data Extraction and Synthesis

For each study, we extracted the following: detection modalities, cognitive states targeted, ML approach, decision framework type, intervention type, sample size, experimental setting, latency, and ecological validity. Risk of bias was assessed across four dimensions. Each dimension was rated as low risk, some concerns, or high risk. The per-study risk of bias ratings have been reported in Appendix A in Table A1. Standard risk of bias tools were not used for the following reasons: Cochrane Risk of Bias 2 assumes a target trial framework that does not apply to engineering validation studies; ROBINS-I is designed for non-randomized intervention studies and lacks domains relevant to sensor-based research, such as signal quality validation and classifier generalization; the Newcastle–Ottawa Scale does not address the specific challenges of real-time physiological computing systems, such as latency reporting and ecological validity. To position the custom framework against an established instrument, we mapped its four domains to the corresponding items of the AXIS appraisal tool for cross-sectional studies. The framework has not been formally benchmarked against AXIS or other instruments, and we acknowledge this as a limitation of our review methodology.

## 3. Results

**Figure 2:**
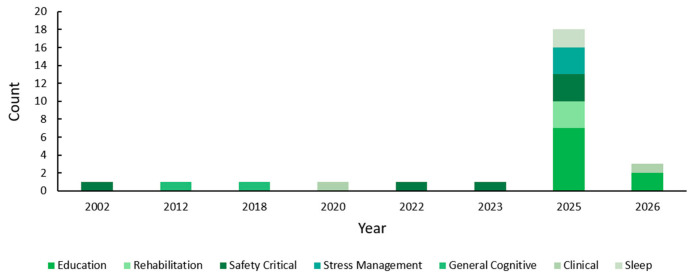
Publication timeline of the studies in the article pool by year (2002–2026).

Recent advances in wearable sensors have led to a rise in research studies regarding using physiological sensors for developing adaptive systems across diverse domains (Figure 2). Applications in education and neurofeedback training accounted for nine studies, while safety-critical domains (driving, aviation, human–robot collaboration) accounted for six despite their greatest potential for improving system safety. Rehabilitation (three studies), clinical (two), sleep (two), stress management (three), and general cognitive (two studies) were the other application domains of such systems.

### 3.1. Sensors for Physiological Measurement

**Figure 3:**
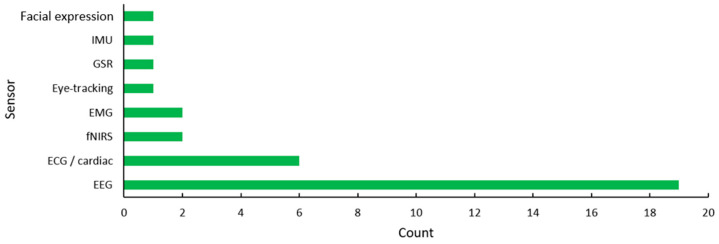
Distribution of sensors within the article pool. Studies employing multiple modalities are counted in each applicable category.

EEG was found to be the most used sensor for detecting cognitive states and was reported in nineteen studies (Figure 3, Table 3). While six studies combined two or more modalities, only one combined EEG and eye tracking [17], achieving 89.3% accuracy and a 27% cognitive load reduction using EEG, ECG, and eye tracking with 173 participants in an Augmented Reality classroom. In contrast, only two studies used Functional Near-Infrared Spectroscopy (fNIRS) [18,19], despite foundational fNIRS work in operator monitoring. Research-grade systems, such as the 64-channel ANT Neuro [17,20], the Brain Products actiCAP at 500 Hz [21,22], and Compumedics Neuroscan amplifiers [23,24], provided high channel counts but required 15–30 min of preparation by trained technicians in controlled electromagnetic environments. On the other hand, consumer-grade devices, such as the OpenBCI Cyton used with dry electrodes [25,26] and a custom single-channel headband [27], were more readily deployable but exhibited degraded contact quality, particularly at temporal and occipital positions where hair impedes electrode contact. Lastly, no study ran the same pipeline with both hardware grades to quantify accuracy degradation.

### 3.2. Signal and Artifact Processing

The findings showed that an EEG was acquired on hardware spanning the research-to-consumer range, with the choice of system reflecting the deployment context of each study. Research-grade montages included the 64-channel ANT Neuro eego system, sampled at 1000 Hz [17] and at 500 Hz [20]; a 32-channel ANT Neuro Waveguard cap, acquired at 2 kHz and downsampled to 100 Hz for model input [28]; Brain Products amplifiers running a 32-channel actiCap at 500 Hz [21,22]; a 64-channel actiCHamp at 500 Hz and downsampled to 125 Hz [29]; a LiveAmp during overnight polysomnography [30]; Compumedics Neuroscan systems, recording 30 [23] and 60 channels [31] at 1000 Hz. Lyu et al. [32] used a 32-channel BioSemi ActiveTwo with active electrodes. Consumer-grade acquisition was dominated by the OpenBCI Cyton, used at 250 Hz for an eight-channel motor-imagery interface [33], a 16-channel dry-electrode cap for a frontal attention index [26], and a 21-channel cap sampled at 125 Hz [25], together with a custom single-channel flexible headband sampling Fp1 and one ECG lead at 512 Hz [27]. One study, instead, used intracranial depth electrodes, recorded at 2 kHz [34].

For EEG-based systems (19/27 studies), seven studies used bandpass filtering, which was most commonly applied in the 1–40 Hz range [23,29,31], with variants of 1–50 Hz [21,35], 1–30 Hz [20,25], and 1–45 Hz [36], each followed by a 50 Hz or 60 Hz notch filter to suppress powerline interference. Filter choices followed the targeted construct: [26] restricted the band to 4–40 Hz because only the beta/(alpha + theta) attention index was required, whereas [30] paired a broad 0.03–80 Hz recording montage with a dedicated 0.25–4 Hz path to capture the slow oscillations that drove the sleep-stage closed loop. One study [25] deliberately omitted the notch filter after confirming an absence of residual line noise, while [28] bypassed band-specific filtering altogether and fed minimally processed broadband data directly to a CNN. Epoch segmentation, likewise, was scaled with the temporal dynamics of the target state, ranging from the 0.5–1 s sliding windows used for fast-updating indices [25,26] to the 10 s epochs used for cognitive flexibility alerts [29] and the 30 s epochs used for fatigue and sleep staging [28,30].

Artifact removal methods varied considerably. Independent Component Analysis was used to remove ocular and muscle components in five studies, in two cases alongside amplitude-threshold epoch rejection, and in one alongside Artifact Subspace Reconstruction [17]; this was feasible only because ICA requires multichannel recordings, which were provided by the research-grade caps used in those studies. Where low channel counts or on-device operation made ICA impractical, lighter approaches were adopted: amplitude-based thresholding on one to a few channels in four studies, blind-source-separation blink removal [21], and wavelet-based baseline-drift removal integrated into the front end of a CNN classifier on a single-channel headband [27]. In [26], motion was additionally detected through an onboard accelerometer in a dry-electrode device. Another study [32], relied on bandpass filtering alone, taking advantage of the high common-mode rejection of its active-electrode BioSemi system.

For cardiac monitoring, used in six studies, preprocessing typically involved R-peak detection followed by RR-interval and heart rate variability extraction. Three studies described their detection method explicitly: Son et al. [37] applied a custom QRS detector with five-min heart-rate averaging; Raggi et al. [38] used the NeuroKit2 toolbox over respiration-gated segments; and [17] used Kubios with automatic artifact correction. The remaining studies bypassed HRV computation. Eye tracking was used for cognitive state detection in only one study [17], which sampled gaze at 1200 Hz and applied a velocity-threshold (I-VT) fixation-detection algorithm. A single study used electrodermal activity [39] that sampled GSR at 10 Hz and computed phasic skin-conductance responses with a rule-based detector, approximating the tonic component with a cumulative response metric rather than formally decomposing the tonic and phasic signals.

Real-time artifact detection and correction were nearly absent from the studies in the article pool. Online rejection or correction during acquisition was performed by only a handful of studies, including the real-time amplitude-threshold gating by [26,30,40], online blink removal by [21], in-line wavelet baseline correction ahead of a CNN by [27], and the post-stimulation refractory period within the closed-loop intracranial platform by [34]. The remaining studies applied preprocessing offline, omitted preprocessing descriptions, or relied on hardware-level filtering alone. Signal processing must handle non-stationary noise, motion artifacts, electrode impedance drift, and electromagnetic interference in real time, yet no study demonstrated a validated real-time preprocessing pipeline that was tested under realistic noise conditions.

### 3.3. Feature Extraction and Classification

A list of findings from all included studies with their detection modalities, cognitive states targeted, classification methods, intervention types, sample sizes, and experimental settings is shown in Table 4, while Table 5 summarizes the performance metrics and latency. EEG frequency bands were the most used in classification systems. Alpha (8–13 Hz) appeared in 12 studies, beta (13–30 Hz) in 9, theta (4–8 Hz) in 10, delta (0.5–4 Hz) in 5, and gamma (>30 Hz) in 4. The beta/(alpha + theta) ratio remained the most common single metric for workload detection. Eye metrics (pupil diameter, fixation duration, blink rate) appeared in one study, and cardiac features appeared in six studies, with five studies using it as the primary modality.

Sixteen studies used an ML approach, and another eight used threshold-based approaches, typically implementing simple rules such as a theta/alpha ratio > 1.2, indicating a high workload. Meanwhile, three studies used statistics or no ML method; thresholds may not generalize across users or sessions. We found that one study tested dynamic versus fixed thresholds and found no difference, suggesting that the limitation may lie in the threshold paradigm itself [21]. The remaining methods included CNN (two studies), SVM (one), k-NN (one), Random Forest (one), state-space model (one), as well as CCA + LDA [32], microstate analysis [20], and a voting classifier with TD3 RL [19]. Among the studies that implemented CNN, one [28] achieved an 88.3% correct intervention application rate, though 11.25% of trials resulted in inopportune stimulation, while the other study [27] demonstrated that deep learning inference is feasible on embedded edge hardware. Classification accuracy, where reported, revealed a wide range; within-subject accuracy ranged from 81.85% [24], to motor imagery at 95.81% [27]. Most studies also reported classification accuracy on the same data used for system operation, potentially inflating performance.

### 3.4. Cognitive States

**Figure 4:**
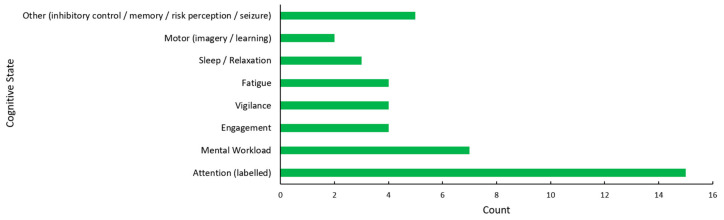
Cognitive states and their proportions across the reviewed article pool.

The distribution of targeted cognitive states is shown in Figure 4 and Table 3. Attention (56%) and mental workload (26%) were most common, with seven studies targeting multiple states. However, only nine studies operationalized a definable attention construct. One study [38] used the State-Trait Anxiety Inventory (STAI), and another [37] used the psychomotor vigilance task (PVT) alongside physiological measures for its measurement. We observed that attention was referred to differently across studies. For instance, one study referred to attention as sustained vigilance [37] when performing a monotonous task over extended periods; another referred to it as targeted attentional engagement [23] in educational settings, using EEG alpha/theta ratios; and another study defined attention [26] via a proprietary ‘attention index’ derived from the eSense algorithm without independent psychometric validation. These three studies would appear identical in a bibliographic search for ‘EEG attention closed-loop systems’, yet they measure different neural substrates and may require different intervention approaches.

### 3.5. Decision Frameworks

A biocybernetics loop (10 studies) is a continuous feedback cycle where the physiological state drives adaptation, while neurofeedback (nine studies) is when physiological signals are continuously monitored to provide audio/visual feedback to the user. The third type was where model-based approaches, such as the CLoSES platform [34] with Bayesian state estimation, were used with trial-by-trial adjustment. Only one study used reinforcement learning (RL) as its decision framework, and [19] used the TD3 RL algorithm, yet the decision framework itself remained rule-based. Most systems implemented binary adaptation (two levels) while only four studies implemented graded adaptation [17,34,42,44]. Interestingly, the dynamic threshold study [21] found that the adaptive system did not improve experience and that the participants reported more negative effects, an important negative result, suggesting that crude binary switching may be more disruptive than helpful.

### 3.6. Interventions and Effectiveness

**Figure 5:**
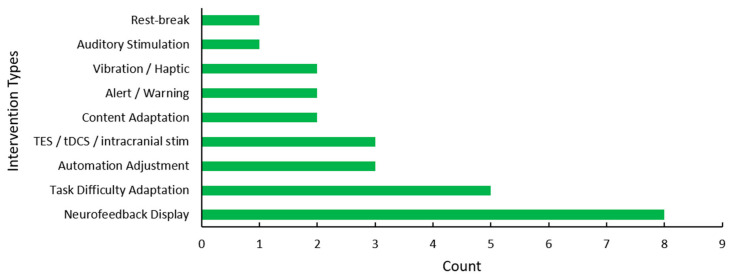
Distribution of intervention types across the 27 included studies. Neurofeedback display (8 studies, 30%) and task difficulty adaptation (5, 19%) were the most common; automation adjustment (3, 11%) was the most operationally relevant but least studied.

We found that automation adjustment appeared in only three studies, while neurofeedback (eight studies) could place the adaptation burden on a user experiencing a high cognitive load (Figure 5). The two studies with the most informative placebo designs found that fake feedback produced comparable improvements to real feedback [25,40]. One study [19] used the passenger’s fNIRS-detected cognitive state to modify autonomous vehicle driving behavior. In rehabilitation domains, a hybrid EMG-EEG system was used to adapt elbow exoskeleton resistance based on detected fatigue [33], and Virtual Reality (VR) was integrated with adaptive hand rehabilitation in another study [42]. These systems serve as examples of the assist-as-needed paradigm [45], implemented through physiological state detection rather than purely kinematic measures.

### 3.7. Latency

The findings show that 9/27 studies (33%) reported a quantified latency, while some described it qualitatively. Among these, reported latencies were 27–681 ms for intracranial stimulation [34], 38 ms for motor rehabilitation [42], <500 ms for elbow rehabilitation [33], and 1.95 s for epilepsy management [44]. Some of the studies that mentioned latency described it qualitatively (e.g., “real-time operation,” “near-instantaneous feedback,” or “sub-second response”) without reporting numerical values. Moreover, even the fastest reported latency was 15 ms, where one study [27], measured the on-device model inference time. Furthermore, all reviewed systems operated reactively, detecting a state after it has occurred and then responding.

### 3.8. Study Type and Sample Size

**Figure 6:**
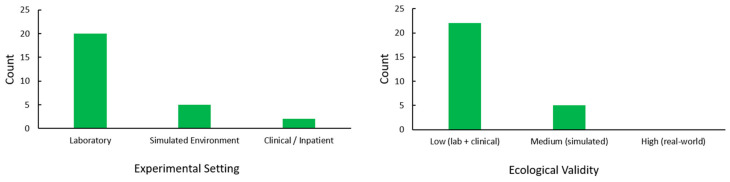
Ecological validity of study settings across the 27 included studies. Laboratory settings dominate (74%), with no study operating in a real-world environment.

A total of 74% of studies operated exclusively in laboratory settings where environmental noise, motion artifacts, and task context were controlled (Figure 6, Table 3). Only one study [44] conducted a field test where an Artificial Intelligence (AI)-based epilepsy management system was used; however, this highlighted the practical challenges of extended-duration monitoring. All 27 studies reported sample sizes, ranging from 3 [29] to 173 [17] and having a median of 20.

## 4. Discussion

### 4.1. Importance of Control Groups and Placebo Conditions in Experimental Designs

Among the reviewed studies, 10 included no control condition, relying entirely on within-subject pre/post comparisons. Of the 17 studies using controls, only 7 used placebo or blinded conditions [20,21,25,30,34,40,46]; the remaining 10 used active controls such as no-intervention baselines, pre/post comparisons with a control task, or non-adaptive system comparisons. The two with the most informative placebo designs produced striking results. For instance, in a study focusing on sham-controlled neurofeedback, in which all 24 participants received fake feedback (where a no genuine neurofeedback condition was included), the researchers found that esthetic design and the illusion of success drove the observed improvements, even without any real neurofeedback signal [25]. On the other hand, another study [40] found that yoked controls (receiving the same difficulty adjustments as the experimental group, untied to their EEG) showed “similar performance” improvements, raising the question of whether periodic task variation alone is enough. Studies targeted neurofeedback specifically, a modality known to be susceptible to placebo and expectancy effects. For example, in a network meta-analysis of 38 ADHD neurofeedback RCTs, the authors found that effect sizes shrank when active controls replaced waitlist controls [47]. Overall, these findings indicate that no study in this article pool provides a non-neurofeedback placebo-controlled test as solid evidence.

### 4.2. Current Limitations in Sensing and Model Performance

The highest classification accuracies were obtained using laboratory-grade EEG with gel electrodes and 32+ channels, yet no study ran the same pipeline with consumer-grade hardware to quantify degradation. The modality-specific artifact resilience documented by [48], where cardiac and eye-tracking signals survived in-flight conditions that rendered EEG unusable, suggests that multimodal architectures may offer a path forward. Furthermore, cross-session, cross-user, and cross-context considerations were missing from the reviewed literature. For instance, it has been demonstrated that individual differences in neurophysiology predict BCI performance, suggesting that cross-user generalization may require per-user adaptation rather than universal classifiers [49]. Active and passive BCI paradigms face distinct transfer challenges [10]. These problems are well-known in the BCI literature [50], but their compound effect on closed-loop systems is overlooked. A system that cannot generalize across sessions, users, and contexts simultaneously requires recalibration at every deployment. Recent EEG foundation models [51,52] pre-trained on large-scale EEG data could potentially address all three simultaneously, yet none have been deployed in a closed-loop system [53].

Reported performance suggests that real-time classification reaches 81.85–95.81% accuracy, although the 95.81% upper bound comes from a small multimodal study. Multimodal sensing improves accuracy ([17], *n* = 173), and edge CNN inference has also been demonstrated [27], along with several informative negative results. Many studies also define cognitive states directly from physiological signals (e.g., an elevated theta as high workload), leading to circular validation where models are tested against the same features used to define the labels. As a result, true performance against independent behavioral or psychometric measures remains largely unknown. Beyond accuracy, the literature does not show that neurofeedback systems outperform placebo (where placebo-controlled data exist), and that models generalize across users or sessions. Additionally, interventions are optimally matched to states, or systems operate outside controlled laboratory settings.

### 4.3. Wearability Considerations and Practical Limitations

Wearable EEG systems offer significant potential for adaptive commercial applications; however, several challenges continue to limit their widespread deployment in real-world environments. One of the primary barriers is maintaining reliable signal quality outside controlled laboratory settings. Commercial users are often required to move freely while working, driving, learning, or interacting with digital systems, introducing motion artifacts, muscle activity, and environmental electrical noise that can substantially degrade EEG recordings and reduce cognitive state classification accuracy [54]. Furthermore, EEG signals exhibit considerable inter-individual variability due to differences in head anatomy, scalp characteristics, hair density, and cognitive responses, often requiring user-specific calibration procedures that increase setup time and reduce scalability. A related challenge is the tradeoff between research-grade and consumer-grade EEG systems. Research-grade devices typically provide high channel counts, wet- or gel-based electrodes, and high sampling rates that support robust neural signal acquisition; however, they generally require 15–30 min of technician-assisted setup, controlled electromagnetic environments, and tethered amplifiers. In contrast, consumer-grade systems can be self-administered within seconds and are substantially more affordable, but they often provide fewer channels, reduced spatial coverage, and poorer electrode contact quality, particularly at temporal and occipital regions where hair interferes with dry-electrode recordings.

Wearability and long-term comfort represent additional barriers to commercial adoption. Adaptive systems frequently require continuous monitoring over extended periods, making ergonomic design critical for user acceptance and compliance. Although dry electrodes improve convenience by eliminating conductive gels, they require sufficient pressure to maintain stable scalp contact, particularly for users with dense or thick hair. Verwulgen et al. found that an average pressure of approximately 12 kPa, corresponding to roughly 72 g of force per electrode, provided an acceptable compromise between comfort and signal acquisition, whereas higher pressures produced significantly greater discomfort [55]. Variations in head size and shape further complicate the design of universal headsets capable of maintaining consistent electrode placement, prompting the development of adaptive geometries that improve fit accuracy by approximately 10–15% compared with conventional designs [55]. Consequently, future adaptive systems will require wearable EEG technologies that preserve research-grade signal quality while remaining ergonomic, affordable, and practical for extended real-world use.

### 4.4. Future Research Directions

#### 4.4.1. Detection of Multiple Cognitive States

While in reality it is typical for multiple cognitive states to co-exist, their interaction has not yet been explored sufficiently in the literature. For instance, a student showing low attention combined with high stress (e.g., test anxiety) would require a different pedagogical support than one showing low attention combined with low arousal (boredom). A single-state attention detection model would provide a similar intervention to both, yet the consequences of that intervention could be fundamentally different.

There were indeed limitations to single-state detection systems that we reviewed. In one study, the target state was accurately detected and stimulation was delivered at the appropriate time; however, memory performance declined in high-ability participants because the system failed to account for arousal-related differences within this subgroup [30]. Similarly, a workload-adaptive system successfully adjusted task difficulty based on detected workload but did not capture the frustration associated with repeated difficulty changes, resulting in increased negative affect [21]. Collectively, these results indicate that monitoring multiple interacting cognitive states could enable more effective and context-appropriate interventions. Although 15 studies targeted “attention,” the construct was defined inconsistently across studies, including sustained attention, selective attention, and attentional engagement. These represent distinct cognitive states with different neural signatures, temporal dynamics, and intervention requirements. Jointly classifying attention type alongside workload and arousal could help distinguish these constructs rather than reducing them to a single label.

##### Modeling for Multi-State Classification of Cognitive States

Recent studies have demonstrated that multi-state classification is technically feasible. One study classified both mental workload and stress from EEG in a passive BCI, achieving 77.5% workload accuracy and 84.1% stress accuracy through transfer learning across 18 participants [56]. Another extended this approach to five co-occurring cognitive states, including cognitive load, distraction, urgency, mind wandering, and interference, using EEG, fNIRS, eye gaze, respiration, and skin conductance data while explicitly modeling interactions among states rather than treating them independently [57]. However, cross-subject accuracy remained modest, suggesting that contextual task information should complement physiological signals. A study based on a multi-label EEG dataset for classifying co-occurring attention states during online learning further demonstrated that multi-label approaches outperform single-label classification [58]. Similarly, cognitive load, affective state, and physiological stress were measured using fNIRS, EEG, ECG, and GSR, revealing that workload increased cognitive load up to a threshold beyond which fNIRS activation declined [59]. Another study developed a forehead-mounted nanomembrane biopatch capable of continuously monitoring drowsiness, cognitive stress, and sleep stage simultaneously, demonstrating the feasibility of multi-state detection in a wearable consumer device [60].

Multimodal fusion can lead to higher model performance. For instance, one study combined EEG and fNIRS data for mental state recognition, achieving F1 scores of 99.36% in subject-dependent settings and 65.05% in subject-independent settings, highlighting the persistent challenge of cross-user generalization as state complexity increases [61]. To support more realistic evaluations, EmoWork was introduced as a multi-label physiological dataset capturing four synchronized affective states of stress, suppression, arousal, and valence, using wearable EEG, ECG, and electrodermal activity (EDA) sensors in interpersonal work scenarios [62]. Finally, a multiple feature block-based CNN simultaneously classified four pilot mental states, fatigue, workload, distraction, and normal, from EEG alone, demonstrating that single-modality data can support multi-state discrimination when paired with appropriate feature engineering [63].

Multi-task learning frameworks have shown strong potential for EEG-based classification. A multi-task CNN with shared temporal and spatial layers simultaneously classified emotion and viewing context from raw EEG, significantly outperforming the single-task EEGNet model and maintaining 91% accuracy at a 10 dB Signal-to-Noise Ratio (SNR), demonstrating that shared feature learning can improve robustness [64]. Similarly, MTEEG, introduced at ICLR 2025, employed a task-agnostic encoder with task-specific low-rank adapters to handle six EEG tasks simultaneously, including abnormality detection, emotion recognition, seizure detection, sleep staging, motor imagery, and event classification. Despite requiring only 1.8M to 7.4M parameters, the model achieved performance comparable to state-of-the-art single-task approaches [65]. A related multi-task emotion recognition framework modeled coupling constraints among valence, arousal, and dominance dimensions, providing a structure that could be adapted for cognitive state assessment by replacing emotion dimensions with workload, fatigue, and stress while enforcing physiologically meaningful coupling constraints [66]. Future studies should first focus on identifying differences between cognitive states, as well as developing and evaluating similar models.

##### Recommendations for Multiple Cognitive State Classification

Detecting multiple states can improve with use of multiple modalities. For instance, EEG measures cortical activity that is related to workload and attention, ECG and heart rate variability (HRV) reflect autonomic arousal associated with stress and fatigue, and eye-tracking captures behavioral indicators such as engagement and vigilance. Combining these signals provides information that cannot be captured by a single modality alone. No single physiological marker is unique to one cognitive state. 

**Figure 7:**
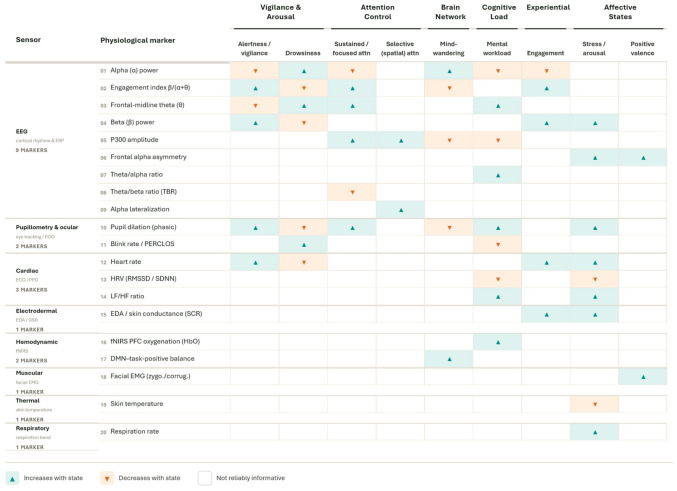
Indicators of change in measures from sensor data per type of cognitive state. Each cell indicates the direction of change expected in the measure as the corresponding cognitive state increases.

As summarized in Figure 7, the same signal typically responds to several states at once: broadly reactive markers such as alpha power and phasic pupil dilation shift across six different states, whereas only a few markers, alpha lateralization, frontal alpha asymmetry, and facial EMG, map onto a single state. Because of this overlap, a rise in frontal-midline theta or a fall in heart-rate variability cannot, on its own, distinguish workload from attention or stress. Reliable real-time detection therefore depends on characteristic combinations across sensor modalities rather than any individual signal, for example, frontal-midline theta increases with posterior alpha desynchronization for sustained attention, or rising skin conductance with falling heart-rate variability for stress [6,67,68]. Different cognitive states leave characteristic signatures across physiological signals, with each modality reflecting a distinct underlying mechanism. For instance, EEG provides the richest set of markers, where oscillatory band power and ratios such as frontal-midline theta, posterior alpha, and the engagement index β/(α+θ) track attention, workload, and alertness, while event-related potentials such as the P300 index the amount of attentional resource allocated to a stimulus [8,68,69]. Autonomic measures complement these, as heart rate variability and the LF/HF ratio reflect sympathetic and parasympathetic balance during stress and fatigue, and electrodermal (skin conductance) activity indexes arousal [69]. Ocular and peripheral signals, including phasic pupil dilation, blink rate or PERCLOS, frontal alpha asymmetry, and facial EMG, add further markers of engagement, drowsiness, and emotional valence [67,70]. Because no single marker is unique to one state (Figure 7), Table 6 lists, for each state, the most discriminating primary marker together with a confirmatory marker from a second modality.

The increased complexity of compound-state classification also requires larger datasets. For example, if workload and fatigue are each classified into three levels, the resulting compound-state space contains nine possible combinations. Furthermore, no study investigated the state transitions of cognitive states (such as alert but bored, mind wandering, microsleep vulnerable). We also observed that no study addressed conflict resolution between two opposing cognitive states. Differences caused by experience levels when working can also lead to different predictions. We recommend that researchers run a closed-loop system that jointly classifies workload, fatigue, and stress as a compound-state vector rather than independent labels, as well as compare intervention outcomes against a single-state classifier on the same participants and task. Subsequently, researchers should train a state-transition model on compound-state trajectories and evaluate whether transition-aware intervention timing outperforms static thresholds. Lastly, researchers should also assess whether systems that adapt to compound states show larger separation from placebo conditions than single-state systems.

#### 4.4.2. Predictive State Trajectories and Intervening Before Decline

Predictive state trajectories may be particularly valuable in bioengineering applications, where intervention timing can directly affect outcomes. In epilepsy management, an implantable device was shown to track patient state over months [44], while predictive seizure forecasting combined with neuromorphic computing achieved more than 97% seizure reduction through proactive stimulation [73]. Together, these findings suggested a clear pathway from long-term monitoring to prediction-driven intervention. A similar opportunity existed for wearable therapeutic systems. Smartwatch-delivered haptic biofeedback was used reactively to manage stress [43], but predictive models could potentially identify escalating stress during extended work periods, such as a 12-h nursing shift, and trigger early interventions before cognitive performance declined. Predictive approaches may also benefit adaptive rehabilitation systems. Existing systems adjusted assistance in response to detected fatigue or performance degradation [33,42]. Forecasting fatigue trajectories could allow assistance to be modified before motor performance deteriorated, enabling proactive rather than reactive support. Moreover, Physiological markers such as increased EEG theta or altered alpha power were detected only after fatigue or vigilance decline had already affected cognitive performance [42]. One study showed that vigilance during monotonous tasks declined gradually over tens of minutes before frequent lapses emerged, with occasional partial recoveries along the way [32]. Such trajectories suggest that predictive systems could identify downward trends and intervene before lapses occur, rather than reacting after performance has already deteriorated.

##### Detection Scale of Models

At the millisecond scale, probabilistic EEG forecasting predicted theta and alpha band activity 150 ms ahead, with approximately 1.0 μV mean absolute error, enabling proactive brain state estimation faster than conventional detection approaches [74]. A predictive-coding-inspired framework, Future-Guided Learning (FGL), used a detection model operating on future data to guide a forecasting model and improved EEG-based seizure prediction performance by 44.8% in terms of AUC-ROC [75]. The millisecond scale, probabilistic EEG forecasting predicted theta and alpha band activity 150 ms ahead, with an approximate 1.0 μV mean absolute error, enabling faster proactive brain state estimation rather than conventional detection approaches [75]. A predictive-coding-inspired framework, Future-Guided Learning (FGL), used a detection model that operated on future data to guide a forecasting model and improved EEG-based seizure prediction performance by 44.8% in terms of the AUC-ROC [76].

At the second-to-minute scale, fatigue trend prediction from EEG was demonstrated using a hybrid CNN, Transformer, and LSTM architecture that forecasted the next time-step fatigue level measured by Percentage of Eye Closure (PERCLOS), moving beyond state recognition toward state prediction [31]. Future cognitive workload was also forecasted from fNIRS data collected during UH-60V helicopter simulation, with LSTM models outperforming CNN-LSTM and Transformer architectures for short-term prediction [76]. A multiview learning framework predicted cognitive load from five modalities, EEG, EDA, ECG, EOG, and eye movements, achieving 81.08% three-class accuracy across 22 participants [77]. Real-time prediction of user errors on a secondary cognitive task was demonstrated using an end-to-end deep learning model [78]. Feedforward EEG forecasting algorithms were also proposed to compensate for hardware and software delays in closed-loop stimulation systems, providing a foundation for anticipatory interventions [79]. In addition, a neuromorphic reservoir computing system forecasted seizure occurrences with 83.33% accuracy and triggered proactive stimulation, achieving more than 97% seizure reduction and demonstrating the potential advantages of prediction-driven interventions over detection-based approaches [73].

At the longitudinal scale, a 10-month study involving 88 participants showed that AI models could forecast changes in cognitive and emotional states from passive smartwatch and smartphone data with an average prediction error of 12.5% [80].

##### Recommendations for Researchers

Short-horizon prediction (<10 s) is most relevant for safety-critical domains such as driving, aviation, and human–robot collaboration. In driving, intervention windows were typically 2–5 s between detectable trajectory change and potential collision, requiring fast-changing signals such as EEG spectral shifts, micro saccades, and steering variability. Probabilistic EEG forecasting at this scale was demonstrated in [74], and Future-Guided Learning [75] showed efficient time-series prediction suitable for real-time deployment [27]. Medium-horizon prediction (<10 min) was relevant for education and training contexts where fatigue and engagement evolved gradually. Ref. [76] demonstrated 3-min-ahead cognitive workload prediction using fNIRS, while existing educational studies in the pool used only reactive detection despite longer session durations (30–90 min) that could have supported predictive adaptation. Long-horizon prediction (hours) captured circadian and shift-work effects. Ref. [81] reported circadian modulation of EEG alpha and theta power, and [80] showed continuous monitoring of digital biomarkers over extended periods using wearable sensors.

We found that no study determined the optimal lead time for proactive intervention, which depends on the cognitive state variations over time, latency of intervention, and the cost asymmetry between false alarms and missed detections. Second, circadian-aware prediction remains unexplored. Cognitive state trajectories follow circadian rhythms that modulate baseline arousal, attentional capacity, and fatigue susceptibility [81]. Referencing signals against an individual’s time-of-day baseline is a complementary mitigation that can help separate endogenous circadian variation from task-induced cognitive decline. Another key limitation is the lack of bidirectional co-adaptation. Although neuromorphic co-adaptive decoders [82] show that hardware can adapt alongside the user, no cognitive state system currently supports mutual learning in which both human and machine continuously adjust their strategies.

## 5. Study Limitations

The article pool was subject to publication and reporting bias. For instance, we found that educational and neurofeedback training settings accounted for 33% of the studies, likely reflecting easier participant recruitment and simpler ethical approval rather than the operational importance of these domains, while safety-critical applications were underrepresented. Future studies should report possible expectancy bias, end-to-end latency (the median and 95th percentile), and conduct multiple sessions that are separated by at least one circadian cycle. Researchers should also include placebo conditions in their experimental designs to develop robust models. We only considered studies that developed real-time adaptive systems rather than specific portions in the development pipeline, which reduced the article pool that was reviewed. Furthermore, no formal certainty-of-evidence assessment was performed, as heterogeneity in outcomes, interventions, and study designs prevented a comparable analysis. The search strategy also prioritized the title, abstract, and author keywords, which may have reduced recall and missed relevant studies. The absence of a formal certainty-of-evidence assessment is a limitation; heterogeneity in outcome measures, intervention types, and study designs across the corpus prevented identifying comparable outcome domains suitable for GRADE; therefore, the strength of the conclusions should be interpreted accordingly. Finally, regulatory considerations for deployment as medical or safety-critical systems, including software as a medical device requirement, were outside the scope of this review and were excluded from the article pool.

## 6. Conclusions

We reviewed 27 studies on real-time adaptive systems for cognitive state detection using physiological signals. EEG was the dominant modality (70%), followed by ECG (22%) and eye-tracking (4%), with 22% of studies using multimodal setups. Attention (56%) and mental workload (26%) were the most common targets, although 15 studies defined “attention” inconsistently across sustained attention, selective attention, and attentional engagement despite their different neural signatures and intervention requirements. No study compared research-grade and consumer-grade hardware using the same pipeline, limiting understanding of deployment feasibility. Future studies should thus also take into consideration wearability factors and the ergonomics of hardware with embedded sensors.

Within-subject classification reached 81.85–95.81% for multi-class tasks in laboratory settings. Most systems relied on static or threshold-based decision rules, and only one study employed reinforcement learning for intervention selection. Neurofeedback display (30%) and task difficulty adjustment (19%) were the most common interventions, while automation adjustment was less frequent (11%). None of the studies demonstrated a clear separation from placebo effects. In addition, 67% did not report end-to-end latency, and no study demonstrated cross-session stability. Overall, most studies (74%) remained at early stages, and none reached field validation. Finally, we identified two key directions: multi-state detection to capture interacting cognitive processes that single-state models missed, and predictive modeling of state trajectories to enable earlier, proactive interventions rather than reactive responses.

## Figures and Tables

**Table 1:** Categorization of different cognitive states and their descriptions.

Category	Description	Cognitive States
Vigilance and Arousal	Regulates global alertness and readiness for processing external stimuli over time	Alert wakefulness; phasic alertness; sustained attention (vigilance); hypoarousal; drowsiness/sleep onset
Attention Control	Mechanisms for selecting, dividing, and regulating task-relevant information	Selective attention; divided attention; executive control
Brain Network States	Large-scale neural configurations supporting internally vs. externally oriented cognition	Task-engaged state; default mode state (DMN); salience switching state
Cognitive Load	Demand on working memory and processing resources relative to capacity	Low load; high load; overload; cognitive fatigue
Experiential States	Subjective quality of engagement and balance between internal and external cognition	Mind wandering; flow state; absorption/immersion
Affective States	Emotional- and arousal-driven influences that bias attention and perception	Stress/high arousal state; hypervigilance

**Table 2:** Database-specific search results.

Database	Records	Search Fields
PubMed	221	Title, Abstract
Scopus	264	Title, Abstract, Author keywords
IEEE Xplore	96	Abstract, Author Keywords, Document Title
Web of Science	97	Title, Abstract, Author keywords
**Total**	**678**	

**Table 3:** Summary of key findings from the review.

Dimension	Outcomes Across Studies from Article Pool	Key Finding
Sample size	Median: 20 participants (range 3–173)	All studies reported sample sizes
Type of studies	Laboratory: 74%; simulator: 19%; clinical: 7%; real world: 0%	Only one study was deployed in an inpatient setting
Sensor types	Electroencephalogram (EEG): 70%; eye tracking: 4%; electrocardiogram (ECG): 22%	22% were multimodal; no study compared research vs. consumer-grade hardware in the same study
Machine Learning classification	ML-based: 59%; threshold-based: 33%; no ML method: 7%	Multi-class accuracy: 81.85–95.81%
Detected cognitive state	Attention: 56%; workload: 26%; fatigue: 15%	“Attention” defined differently across 15 studies; no study modeled state interactions
Decision framework	Biocybernetic loop: 10; neurofeedback: 9; model-based: 8	One study uses reinforcement learning (RL) for the decision framework; all use static or threshold-based logic
Intervention type	Neurofeedback (NFB) display: 30%; task difficulty: 19%	Automation adjustment in only 11% of studies
Latency of output	15 ms–2.5 s	Nine studies reported end-to-end latency, and all systems were reactive

**Table 4:** List of studies with detection modalities, cognitive states targeted, classification methods, intervention types, sample sizes, and experimental settings.

Article	Year	Sensor Type	Cognitive States	Machine Learning Approach	Intervention	Sample Size	Setting
[40]	2002	EEG (4 channel)	Vigilance, engagement	Threshold-based	Task difficulty adaptation	40	Laboratory
[41]	2012	EEG	Engagement, mental workload	-	Task difficulty adaptation	20	Laboratory
[21]	2018	EEG, facial tracking	Cognitive load, emotional valence	Threshold-based	Task difficulty adaptation	16	Laboratory
[34]	2020	iEEG	Cognitive flexibility	State-space model	Intracranial direct simulation	12	Clinical
[32]	2022	EEG (32 channels)	Vigilance, attention (sustained attention)	CCA + LDA	Automation adjustment	17	Laboratory
[28]	2023	EEG (32 acquired, 14 used), ECG	Vigilance, attention (sustained attention), fatigue	CNN	TES/tDCS	6 used from sample of 19	Laboratory
[33]	2025	EMG, EEG	Fatigue	SVM + k-NN + Bayesian fusion	Automation adjustment	5	Laboratory
[42]	2025	EMG, IMU	Motor learning, fatigue	Random Forest + SVR	Task difficulty adaptation	24	Laboratory
[22]	2025	EEG (32 channel)	Attention (general),	-	Content adaptation	65	Laboratory
[30]	2025	EEG	Memory/sleep consolidation	Threshold-based	TES/tDCS	20	Laboratory
[27]	2025	EEG (single channel) + single channel ECG	Attention (generic/attention deficit)	CNN	Neurofeedback display	18	Laboratory
[29]	2025	EEG (64 channel)	Fatigue	TSMNet	Alert/warning	3	Simulated environment
[35]	2025	EEG	Attention, mental workload	Threshold-based	Neurofeedback display	60	Simulated environment
[43]	2025	HR/ECG	Relaxation/sleep onset	Threshold-based	Haptic biofeedback	20 + 28	Laboratory
[25]	2025	EEG (21 channel cap)	Workload/motivation	-	Neurofeedback display	24	Laboratory
[38]	2025	ECG + respiration	Stress	Random Forest regressor	Auditory stimulation	13	Laboratory
[37]	2025	ECG	Sleep inertia	Threshold-based	Vibration/haptic	15	Laboratory
[26]	2025	EEG (16 channel)	Attention (sustained), engagement	Threshold-based	Neurofeedback display	30	Laboratory
[39]	2025	GSR	Cognitive load	Threshold-based	Rest break trigger	36	Laboratory
[23]	2025	EEG (30 channel)	Attention (sustained), engagement	k-NN	Task difficulty adaptation	19	Laboratory
[24]	2025	EEG (60 channel, 36 used)	Motor imagery	OVR-CSP + SVM	Neurofeedback display	48	Simulated environment
[18]	2025	fNIRS (35 channel)	Inhibitory control	MVPA decoder	Neurofeedback display	45	Laboratory
[17]	2025	EEG (64 channel), ECG, eye-tracking	Attention (sustained attention)	- (89.3% acc.)	Content adaptation	173	Simulated environment
[19]	2025	fNIRS	Risk perception	Voting classifier + TD3 RL	Automation adjustment	10	Simulated environment
[20]	2026	EEG (64 channel)	Attention (sustained attention)	Microstate analysis	Neurofeedback display	19	Laboratory
[44]	2026	EEG	Epilepsy	Deep learning	alert/warning	13	Inpatient
[36]	2026	EEG	Gamma neuromodulation	Unsupervised clustering	Neurofeedback display	31	Laboratory

**Table 5:** Classification approaches, their performance, and latency differences.

Citation	Year	Machine Learning Approach	Accuracy/Key Metric	Latency
[34]	2020	State-space model	Trial-by-trial state estimation	27–681 ms
[32]	2022	CCA + LDA	88%	-
[28]	2023	CNN	88.3% correct intervention application	-
[33]	2025	CSP-SVM + kNN + Bayesian fusion	94.5% (intent detection) 88.2% (fatigue)	<500 ms
[42]	2025	Random forest	R^2^ = 0.72; SUS = 84	38 ms
[23]	2025	k-NN	87% (attention classification)	-
[17]	2025	Ensemble (SVM/CNN/RF)	89.3% (four-way disorder classification)	-
[19]	2025	Voting Ensemble (+TD3 RL)	0.77 balanced accuracy	
[44]	2026	Deep learning (CNN/LLM)	Longitudinal co-management	1.95 s
[27]	2025	CNN	95.81%	15.18 ms
[26]	2025	None (threshold-based)	NA	128 ms
[35]	2025	None (threshold-based)	NA	200 ms
[20]	2026	None (threshold-based)	NA	250 ms/iteration
[24]	2025	Filter bank OVR-CSP + SVM	81.85%	2.5 s

**Table 6:** Recommended markers from physiological sensor data for detecting cognitive states. Each primary marker changes as the state intensifies.

Cognitive State	Recommended Marker	Indicator of Measure	Reference
Alertness/vigilance	Engagement index β/(α + θ)	Beta increase, alpha decrease	[8]
Drowsiness	PERCLOS (eye closure)	Alpha and theta increase, heart rate decreases	[67]
Sustained/focused attention	Frontal-midline theta	Posterior alpha desynchronization, P300 increase	[68]
Selective (spatial) attention	Posterior alpha lateralization	P300 increase to the attended side	[71]
Mind wandering	DMN–task-positive balance	P300 decrease (perceptual decoupling)	[72]
Mental workload	Theta/alpha ratio	Frontal-midline theta increase, parietal alpha decrease, pupil dilation increase	[67]
Engagement	Engagement index: β/(α + θ)	Heart rate increase, skin conductance (SCR) increase	[8]
Stress/arousal	EDA/skin conductance (SCR)	Heart rate variability decrease (LF/HF increase)	[69]
Positive valence	Frontal alpha asymmetry (left > right)	Facial EMG increase	[70]

## Data Availability

No new data were created or analyzed in this study. Data sharing is not applicable to this article.

## Supplementary Materials

The following supporting information can be downloaded at: https://www.mdpi.com/article/10.3390/bioengineering13070734/s1.

## Abbreviations

The following abbreviations are used in this manuscript:

AI	Artificial Intelligence
AR	Augmented Reality
AUC-ROC	Area Under the Receiver Operating Characteristic Curve
BCI	Brain–Computer Interface
CLoSES	Closed-Loop System for Electrical Stimulation
CNN	Convolutional Neural Network
CONSORT	Consolidated Standards of Reporting Trials
DBS	Deep Brain Stimulation
ECG	Electrocardiography
EDA	Electrodermal Activity
EEG	Electroencephalography
EMG	Electromyography
EOG	Electrooculography
fNIRS	Functional Near-Infrared Spectroscopy
GSR	Galvanic Skin Response
HR	Heart Rate
HRV	Heart Rate Variability
ICA	Independent Component Analysis
IMU	Inertial Measurement Unit
k-NN	k-Nearest Neighbors
LF/HF	Low Frequency/High Frequency ratio
LSTM	Long Short-Term Memory
ML	Machine Learning
NFB	Neurofeedback
PERCLOS	Percentage of Eye Closure
PRISMA	Preferred Reporting Items for Systematic Reviews and Meta-Analyses
PROSPERO	International Prospective Register of Systematic Reviews
PVT	Psychomotor Vigilance Task
RL	Reinforcement Learning
RMSSD	Root Mean Square of Successive Differences
SDNN	Standard Deviation of NN intervals
SNR	Signal-to-Noise Ratio
STAI	State-Trait Anxiety Inventory
SVM	Support Vector Machine
tDCS	Transcranial Direct Current Stimulation
TES	Transcranial Electrical Stimulation
TRL	Technology Readiness Level
TRIPOD	Transparent Reporting of a Multivariable Prediction Model for Individual Prognosis Or Diagnosis
VR	Virtual Reality
XR	Extended Reality

## Appendix A

Risk of bias was assessed independently by both authors (A.R.K. and P.M.K.). Because the included studies employed diverse designs (proof-of-concept demonstrations, within-subjects experiments, controlled evaluations, and case studies) that do not align with a single standardized tool, a custom assessment framework was developed. Cochrane RoB 2 is designed for randomized controlled trials and assumes a target trial framework that does not apply to engineering validation studies. ROBINS-I is designed for non-randomized studies of interventions and lacks domains relevant to sensor-based research, such as signal quality validation and classifier generalization assessment. The Newcastle–Ottawa Scale, while applicable to observational studies, does not address the specific methodological challenges of real-time physiological computing systems, such as latency reporting and ecological validity of the sensing environment. Accordingly, a four-domain framework was developed to capture the most relevant sources of bias in this literature. Each domain was rated as low risk (+), unclear risk (?), or high risk (-) of bias. Disagreements between reviewers were resolved through discussion. Inter-rater agreement for the risk of bias assessment yielded Cohen’s κ = 0.83 across all four domains. Evidence strength was evaluated narratively based on the consistency of results, study quality per the framework, and the size and diversity of the supporting evidence base.

Domain definitions: B1 (Randomization and Blinding) was rated high risk (-) if no randomization or blinding was reported. B2 (Sample Size Adequacy) was rated low risk (+) if N ≥ 20, unclear (?) if the sample size was not reported, and high risk (-) if N < 20. No study reported a priori power analysis. B3 (Control Condition) was rated high risk (-) if no control or sham condition was used, low risk (+) if sham or blinded control was included. B4 (Outcome Reporting) was rated high risk (-) if classification accuracy, latency, or effect sizes were omitted, low risk (+) if reported. B4 was rated unclear (?) when studies reported partial outcome data (e.g., qualitative performance descriptions without quantified accuracy or effect sizes). Overall risk was aggregated as follows: low risk (+) if 0 high-risk domains, unclear risk (?) if 1–2 high-risk domains, and high risk (-) if 3–4 high-risk domains.

**Table A1:** Per-study risk of bias ratings across four domains: (+) = low risk, (?) = unclear risk, and (-) = high risk.

Citation	Year	B1: Randomization	B2: Sample Size	B3: Control	B4: Reporting	Overall
[40]	2002	-	+	+	?	?
[41]	2012	-	+	-	-	-
[21]	2018	-	+	+	?	?
[34]	2020	-	+	+	+	?
[32]	2022	-	+	-	+	-
[28]	2023	-	+	-	+	-
[33]	2025	-	+	-	+	-
[42]	2025	-	+	-	+	?
[20]	2026	+	+	+	+	+
[22]	2025	-	+	-	-	-
[44]	2026	-	+	-	-	-
[30]	2025	-	+	+	+	?
[27]	2025	-	+	-	+	?
[29]	2025	-	+	-	-	-
[36]	2026	+	+	-	-	-
[35]	2025	-	+	-	?	?
[43]	2025	-	+	-	+	?
[25]	2025	-	+	+	?	?
[38]	2025	-	+	-	+	?
[37]	2025	-	+	-	+	?
[26].	2025	-	+	-	?	?
[39]	2025	-	+	-	?	?
[23]	2025	-	+	-	+	?
[24]	2025	-	+	-	-	-
[18]	2025	+	+	-	?	?
[17]	2025	-	+	-	+	?
[19]	2025	-	+	-	-	-
