# Supplementary material for: Detecting and Improving Human Cognitive State in Real-Time Using Data-Driven Adaptive Systems: A Systematic Review
Source: Bioengineering (Basel). 2026 Jun 24;13(7):734. doi: 10.3390/bioengineering13070734 (PMC13406092; doi:10.3390/bioengineering13070734)
Supplement: Supplementary file 1 [file bioengineering-13-00734-s001.zip › bioengineering-4342664-supplementary.pdf]

Supplementary Material S1. Database-specific search strategies

Search date: March 2026. Four databases were searched: PubMed, Scopus, IEEE Xplore, and Web of Science. The same three-concept Boolean logic was applied across all databases and adapted to each database's field-tag syntax. Search terms were restricted to title, abstract, and author-keyword fields (field availability differs by database, as noted below).

1. Conceptual search logic

("closed-loop" OR "biocybernetic" OR "neuroadaptive" OR "neurofeedback" OR "biofeedback" OR "brain-computer interface" OR "adaptive automation") AND

("EEG" OR "fNIRS" OR "ECG" OR "eye tracking" OR "EMG" OR "heart rate variability" OR "GSR") AND

("cognitive state" OR "mental workload" OR "cognitive load" OR "attention" OR "fatigue" OR "engagement" OR "vigilance" OR "stress")

2. Field mapping and record counts by database

Database	Search fields	Field syntax	Records
PubMed	Title, Abstract		221
Scopus	Title, Abstract, Author Keywords	TITLE-ABS-KEY( )	264
IEEE Xplore	Document Title, Abstract, Author Keywords	"Document Title": / "Abstract": / "Author Keywords":	96
Web of Science	Title, Abstract, Author Keywords	TS= (Topic)	97
**Total**			**678**

No language, date, or document-type filters were applied at the search stage; eligibility filtering was performed during screening per the criteria in Section 2.2.

3. PubMed

("closed-loop" OR "biocybernetic" OR "neuroadaptive" OR "neurofeedback" OR "biofeedback" OR "brain-computer interface" OR "adaptive automation") AND ("EEG" OR "fNIRS" OR "ECG" OR "eye tracking" OR "EMG" OR "heart rate variability" OR "GSR")
AND ("cognitive state" OR "mental workload" OR "cognitive load" OR "attention" OR "fatigue" OR "engagement" OR "vigilance" OR "stress")

4. Scopus

TITLE-ABS-KEY("closed-loop" OR "biocybernetic" OR "neuroadaptive" OR "neurofeedback" OR "biofeedback" OR "brain-computer interface" OR "adaptive automation") AND TITLE-ABS-KEY("EEG" OR "fNIRS" OR "ECG" OR "eye tracking" OR "EMG" OR "heart rate variability" OR "GSR") AND TITLE-ABS-KEY("cognitive state" OR "mental workload" OR "cognitive load" OR "attention" OR "fatigue" OR "engagement" OR "vigilance" OR "stress")

5. IEEE Xplore

(“closed-loop" OR "biocybernetic" OR "neuroadaptive" OR
"neurofeedback" OR "biofeedback" OR "brain-computer interface" OR
"adaptive automation")
AND
("EEG" OR "fNIRS" OR "ECG" OR "eye tracking" OR "EMG" OR
"heart rate variability" OR "GSR")
AND
("cognitive state" OR "mental workload" OR "cognitive load" OR
"attention" OR "fatigue" OR "engagement" OR "vigilance" OR "stress")

6. Web of Science

TS (Topic) searches title, abstract, author keywords, and Keywords Plus.

TS=("closed-loop" OR "biocybernetic" OR "neuroadaptive" OR "neurofeedback" OR "biofeedback" OR "brain-computer interface" OR "adaptive automation") AND TS=("EEG" OR "fNIRS" OR "ECG" OR "eye tracking" OR "EMG" OR "heart rate variability" OR "GSR") AND TS=("cognitive state" OR "mental workload" OR "cognitive load" OR "attention" OR "fatigue" OR "engagement" OR "vigilance" OR "stress")
